# Influence of engine heat source conditions on a small-scale CO_2_ power generation system

**DOI:** 10.1016/j.fmre.2023.05.002

**Published:** 2023-05-15

**Authors:** Ligeng Li, Hua Tian, Xin Lin, Xianyu Zeng, Yurong Wang, Lingfeng Shi, Xuan Wang, Xingyu Liang, Gequn Shu

**Affiliations:** a State Key Laboratory of Engines, Tianjin University, Tianjin 300072, China; b Department of Thermal Science and Energy Engineering, University of Science and Technology of China, Hefei 230027, China

**Keywords:** Power generation, Supercritical CO_2_, Turbine expander, Printed circuit heat exchanger, Engine waste heat recovery

## Abstract

Employing a CO_2_ power generation system to recover waste heat from engines can reduce fuel consumption and CO_2_ emissions by producing additional electric power. Nevertheless, the fluctuation in engine operating conditions would cause variations in waste heat sources and affect system performances largely. Hence, an experimental performance test at various engine conditions was implemented by the construction of a small-scale (10 kW) CO_2_ power generation system. Key components, including the turbine expander and printed circuit heat exchanger, were specifically designed and constructed. The steady-state and transient performances of critical components and the integrated system were carried out. Experimental results of the turbine expander at varying engine conditions revealed the potential for long-term and stable operation under dynamic mass flow rate, inlet temperature, and pressure ratio. The maximum total generation power and efficiency reached 11.55 kW and 58.92%. The printed circuit heat exchanger used to exploit engine exhaust gas showed satisfactory performances in balancing the trade-off between heat transfer and pressure drop. The total pressure drop of engine exhaust gas was lower than 4 kPa determined by both exhaust mass flow and temperature, considering all the variable engine conditions. Despite that a performance penalty was observed at the off-design operation of the integrated system because of the decrease in the waste heat input, the maximum net power and thermal efficiency reached 10.57 kW and 6.59%, respectively, at the engine condition of 1100 rpm, 1200 N m, with a relative improvement of 6.3% in engine brake thermal efficiency.

## Graphical abstract

**Figure:**
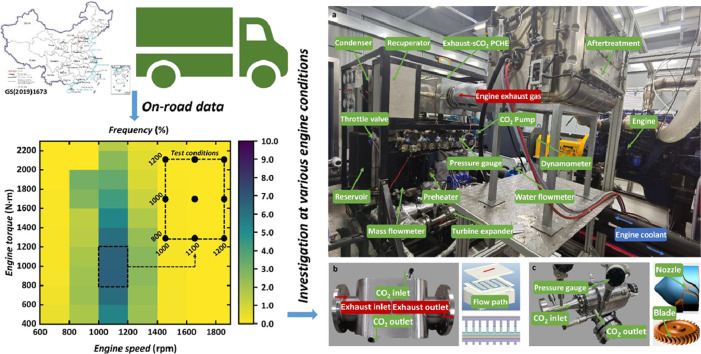

## 1 Introduction

Climate change has a great impact on human societies and may provoke subsistence crises and civilizational collapses among human societies [1]. Global mean surface temperature is increasing at the rate of 0.2 ± 0.1 °C per decade which may cause major implications for multiple geographies, climates, and ecosystems [2]. In 2015, the Paris Agreement reached a breakthrough in reducing global greenhouse gas (especially CO_2_) emissions to limit the global temperature increase to 2 °C even further to 1.5 °C [3]. The CO_2_ utilization is considered an effective method to reduce CO_2_ emission, as reported by Irwin et al. [4], which makes the technology of non-conversion CO_2_ use a research hotspot. According to the report by IEA, CO_2_ can be used directly as a heat transfer fluid in power cycle systems to contribute to climate goals [5]. The power cycle with the working fluid of CO_2_ has been studied widely in many application areas such as fossil fuel, waste heat, concentrated-solar power, nuclear power, and geothermal power [6]. High efficiency, economy, and compactness are its advantages and therefore attract a lot of attention worldwide [7].

The CO_2_ transcritical power cycle (CTPC), one of typical thermodynamic cycles, shows great advantages compared with the supercritical CO_2_ power cycle in compression power consumption because the compression process is closer to the saturated liquid line. For instance, when the system mass flow rate is set to be 1.0 kg/s, and both the compressor and the pump are operated at the same conditions of 7.4 MPa inlet pressure and 20 MPa outlet pressure, the input power for the CTPC is decreased by 40.4% from 34.68 kW to 20.67 kW compared with the supercritical CO_2_ (sCO_2_) power cycle [8]. In addition, the size of the compressor is larger than that of the pump at the same outlet condition as there is a large density difference near the critical point, which is important for application scenarios with limited space [9]. Regarding the heating process, the working fluid in a CTPC system is heated directly from the liquid state into the supercritical state, which has considerable potential in reducing exergy loss because of a better temperature matching between the heat source and the working fluid [10]. Because of the foregoing, the CTPC is more suitable for the application area where the space is relatively limited, the power output is required, and the heat source should be used as much as possible.

Hence, several studies have indicated that it is an effective way to recover part of the electric power and to reduce the fuel consumption and CO_2_ emission to the atmosphere by applying the CTPC to a distributed energy system or engine waste heat recovery in transportation areas. Engines are the prime movers in distributed energy systems and are still the main power source in the transportation area, and the space in a building-level distributed energy system or a vehicle is insufficient. Additional net power output will lead to an improvement in engine brake thermal efficiency (BTE), which has great potential for fuel saving and greenhouse gas emission reduction [11]. Many researchers conducted parameter optimization to instruct the system operation. Sadreddinia et al. [12] and Li et al. [13] compared the thermodynamic performances of their proposed CTPC systems in waste heat recovery. Nevertheless, both the temperature and mass flow rate of the heat source are kept the same. In addition to thermodynamic analysis, Zhou et al. [14] and Zhang et al. [15] focused on thermo-economic analysis when the waste heat from a natural gas engine is recovered mainly. Multiobjective optimization based on the genetic algorithm is carried out for both studies. These studies are on the fundamental of a constant waste heat source. In addition, many studies laid stress on configuration optimization to improve the thermal efficiency of the CTPC [16]. Zhi et al. [17] and Wang et al. [18] adopted layouts of the parallel cycle and the multi-expansion cycle in CTPC to adapt to multiple engine waste heat sources and improve the recovery of exhaust gas. The isentropic efficiencies of both the pump and turbine are set to be constant [17,19].

Loads of previous studies pay attention to the design and optimization at the design condition where a fixed heat source of engines is adopted, and their focus remains on the modeling which is based on assumed isentropic efficiency, pinch point temperature or effectiveness, and heat transfer correlations. Nevertheless, a small-scale flexible generation system for engine waste heat recovery or distributed energy resource is prone to operate under off-design conditions [20]. Unlike most thermal power plants performing grid load-following functions, the power generation system in the engine area should be encouraged to perform source-following ability. Namely, the net power output should be improved not only at a fixed engine condition, but the ability to follow the variation of the heat source is also vital as a response to varying engine waste heat conditions caused by the large distributed engine operational profile. Taking a diesel engine with a rated power of 294 kW as an example, the maximum waste heat reaches 230 kW at the rated engine condition, which is almost 200 kW higher than that of the idling condition mainly because of larger drops in both waste heat mass flow rate and temperature [21].

Hence, some study focused on the off-design modeling and estimation. Concerning the turbomachinery modeling, limited by the insufficient experimental data, Dyreby [22] summarized 4th polynomial fits of the modified non-dimensional head-flow and efficiency-flow curves from the radial compressor test results reported by Sandia National Lab [23]. The turbine efficiency was scaled with the operating efficiency including the aerodynamic losses also in form of 4th polynomial fits as a function of the velocity ratio [22]. In addition, the loss models and CFD methods are also effective approaches to figuring out turbomachinery off-design performances [24,25]. Regarding the heat exchanger off-design modeling, it is usually assumed that the fluid properties are constant and the heat transfer coefficient is proportional to the mass flow rate at off-design conditions, which is mainly determined by the exponent of Reynolds number when calculating the Nusselt number [26].

Although there are many correction models, the investigation has not yet been carried out on the selection of the best-suited models for CO_2_ turbomachinery applications especially applied to small-scale power generation systems because the experimental data of the turbomachinery in a supercritical CO_2_ environment is limited [27]. Meanwhile, it should be noted that most experimental results mainly focused on the power generation system of MW or even 10 MW scale. Scarce attention is paid to the design and experimental tests of a small-scale (∼10 kW) power generation system where the heat source is within the range of 100–500 kW, which is provided by the engine in the transportation area and distributed energy resource. Another shortcoming is that when the engine operated at off-design conditions, how much influence the waste heat recovery system would have on the original engine. On the one hand, changes in both mass flow rate and temperature of engine exhaust gas will contribute to the variation in the pressure drop produced in the heat exchanger, which may cause slight engine power loss. Hence, from the point of the heat exchanger design, not only a sufficient heat transfer should be achieved but optimization should be carried out to decrease the pressure drop as much as possible. On the other hand, the operation of the sCO_2_ turbomachinery in various heat source conditions has a great impact on system performance. Performances including isentropic or total generation efficiency, bearing, and stator winding operating states should be investigated to instruct the improvement in turbomachinery design. In addition, although plenty of studies have provided the thermodynamic performance of the power generation itself, performance evaluation including the energy-saving potential of the original engine is still insufficient to figure out how much improvement the power generation system can bring about for the original engine.

The present work aims to implement an experimental test at various engine conditions by the construction of a small-scale CO_2_ power generation system, and to reveal the steady state and transient performances of both key components and the integrated system. The introduction is followed by a section about test facilities. After the developed key components including the turbine expander and the printed circuit heat exchanger (PCHE) are explained, the filling system used to charge the working fluid and the data acquisition system are also described. Based on a large number of road load data, the common 9 engine working conditions are outlined before results for detailed transient and steady performance evaluation for both key components and the integrated system are given to assess the source-following performances. Then the energy-saving potential of the constructed CO_2_ power generation system is summarized. The last section consists of conclusions and the next steps.

## 2 Test facilities

### 2.1 Engine-CTPC system

**Fig. 1:**
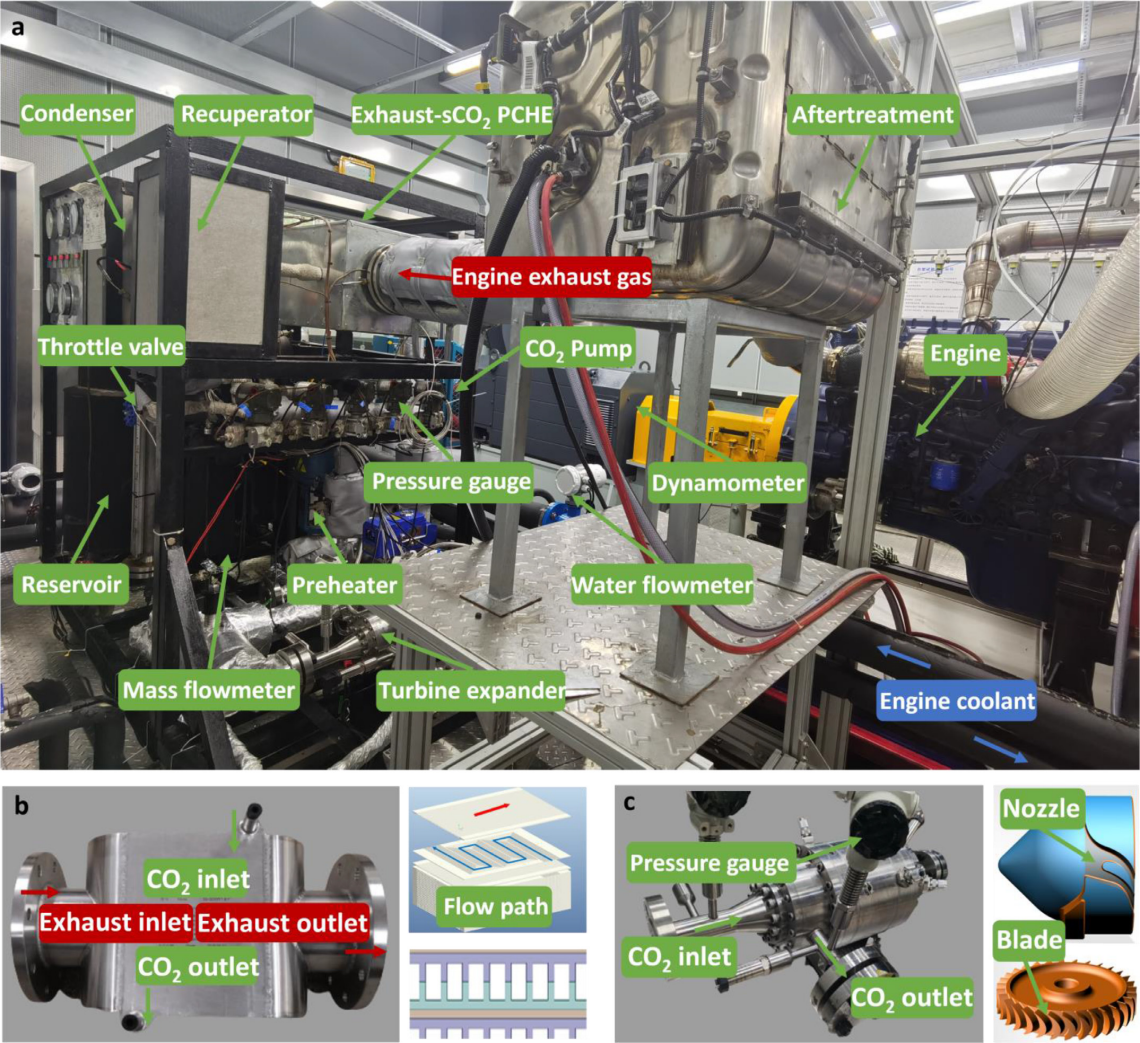
**Photo of the engine-CTPC test system**. (a) Engine-CTPC test bench. (b) Photo and schematic diagram of the exhaust-sCO_2_ PCHE. (c) Photo and schematic diagram of the TG system.

**Fig. 2:**
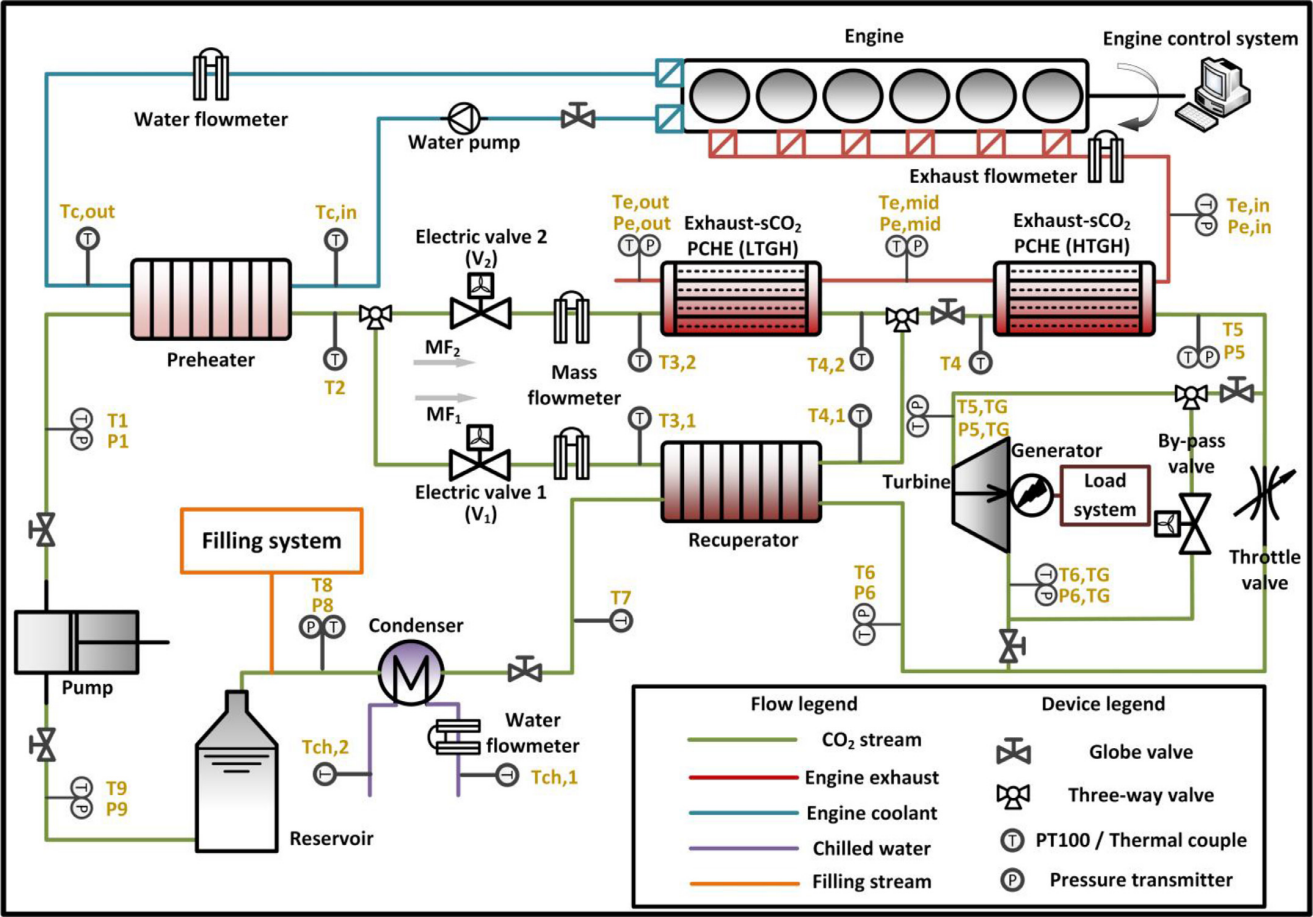
**Schematic diagram of the CTPC system**.

The photo and the schematic diagram of the engine-CTPC system are shown in Fig. 1, Fig. 2, respectively. In this study, an inline four-stroke six-cylinder diesel engine was used as the target engine to provide waste heat sources including the engine exhaust and coolant, which are considered the most promising waste heat sources in engine waste heat recovery because nearly 50% of the thermal energy combusted from the fuel is emitted through these two waste heat sources. Detailed parameters are listed in Table 1. Insulation measures should be taken to prevent heat transfer to the ambient. Blue and red solid lines indicate the stream of engine coolant and exhaust, respectively. A standard engine measurement and control system was equipped to ensure stable operation and precise data acquisition.

**Table 1:** **Specifications of the engine used in this study**.

Properties	Values	Units
Type	In-line, 6-cylinder, 4-stroke	–
Air intake mode	Charge inter-cooling	–
Valve number	4	–
Total displacement	12.5	L
Rated speed	1900	rpm
Maximum torque	2300	N m
Rated power	360	kW

As the key component to transfer the thermal energy into mechanical work, the turbogenerator (TG) system was designed to include a partial-admission turbine expander, a high-speed synchronous generator, revolution speed, pressure and temperature sensors, and valves. Concerning the pipe design, a three-way valve was used to determine the working fluid streaming through the turbine or bypass branch in case the working condition of the turbine is deteriorating such as overpressure, overspeed, or vibration alarm. In addition to the safety issue, a resisting gas was designed from the rear of the generator considering the sealing and cooling problem. It should be noted that the operation of the TG may encounter significant problems, including the sealing and cooling because the high-temperature and high-pressure working fluid in the turbine may escape into the generator chamber, potentially damaging the generator and reducing its efficiency. In this study, the resistant gas was introduced from the high-pressure side working fluid after the preheater. The pressure of the CO_2_ could be reduced to an appropriate pressure that is slightly higher than that of the working fluid after the expansion to avoid leakage into the generator chamber. Meanwhile, the resistant gas could also achieve heat dissipation of the generator in case the coil is burned out. The rotational speed of the turbine is usually extremely high because the mass flow rate of the CO_2_ is low but its density is high at a supercritical state considering the property of the CO_2_. This will lead to unavoidable secondary flow loss. Hence, we think that it is practicable to adopt a partial-admission aerodynamic configuration with a 6% admission rate to reduce the secondary flow loss. The nozzle has two symmetrical flow channels to accelerate the sCO_2_. The mean diameter of the rotor is 26 mm with a blade height of 4 mm. The generator was integrated with the turbine coaxially to transform the mechanical work into electric power, which could be consumed by the TG load system consisting of eight resistors in parallel (introduced in Section 2.3).

For CO_2_ power cycles, the waste heat utilization rate and physical size are significantly affected by the gas heater, where the engine exhaust gas is recovered. The design requirements include enduring the working conditions of high pressure and high temperature, enduring particulate matter from the exhaust, ensuring heat transfer, and decreasing the dimensions, investment, and maintenance costs as much as possible. In this study, two PCHEs for exhaust-sCO_2_ gas heaters were developed and used to absorb heat from the engine exhaust. Both the working fluid and the engine exhaust flow through the straight channels because complicated channels such as zigzag and S-shaped channels will increase the exhaust pressure drop leading to power loss of the original engine although the heat transfer may be enhanced. A single flow area of the engine exhaust channel reaches 6 mm^2^, to match the required exhaust pressure drop constraints. The single flow area for the sCO_2_-side channel is 2 mm^2^. The core of the heat exchanger and shell were both manufactured based on the material UNS-S30408.

The CTPC system was constructed and integrated as the bottom cycle. The green solid line represents the CO_2_ stream. Firstly, the liquefied CO_2_ is pressurized by the pump after it is sucked from the reservoir. Secondly, the CO_2_ streams through the preheater, where the working fluid is heated by the engine coolant. Subsequently, it is separated into two branches. One of the branches absorbs waste heat from the low-temperature exhaust in a low-temperature gas heater (LTGH). A recuperator was installed in the other branch. After their mixing, the CO_2_ enters the high-temperature gas heater (HTGH), where the high-temperature engine exhaust is recovered. Third, the high-pressure and high-temperature working fluid produces the work in the TG system. Finally, the CO_2_ streams through the recuperator and is condensed in the condenser and finally flows back into the reservoir. Indispensable valves were specifically designed, including two electric valves, a throttle valve, and a bypass valve. Electric valves were installed in both splitting branches to independently control the mass flow. The throttle valve is used when the TG system is not operating. The bypass valve in the TG system was set to ensure the safe operation of the TG under abnormal scenarios. The technical specifications of the main components are presented in Table 2.

**Table 2:** **Specifications of the main components**.

Component	Item	Specification
Pump	Type	Reciprocating plunger pump
	Rated flow rate	4 m^3^/h
	Maximum pressure	16 MPa
Expander	Type	Partial admission axial turbine
	Designed electric power	13.7 kW
	Maximum speed	40,000 rpm
Generator	Type	Synchronous motor
	Connection type	Coaxial
Preheater	Type	Plate
	Area	6.81 m^2^
	Flow type	Counter-current
Recuperator	Type	Plate
	Area	9.08 m^2^
	Flow type	Counter-current
LTGH	Type	PCHE
	Area	3.00 m^2^
	Size	240 × 150 × 166.3 (mm)
	Flow passages	Straight channel
HTGH	Type	PCHE
	Area	5.97 m^2^
	Size	310 × 240 × 190 (mm)
	Flow passages	Straight channel
Condenser	Type	Plate
	Area	5.90 m^2^
	Flow type	Counter-current
Reservoir	Type	Vertical pressure vessel
	Volume	40 L

### 2.2 Filling system

**Fig. 3:**
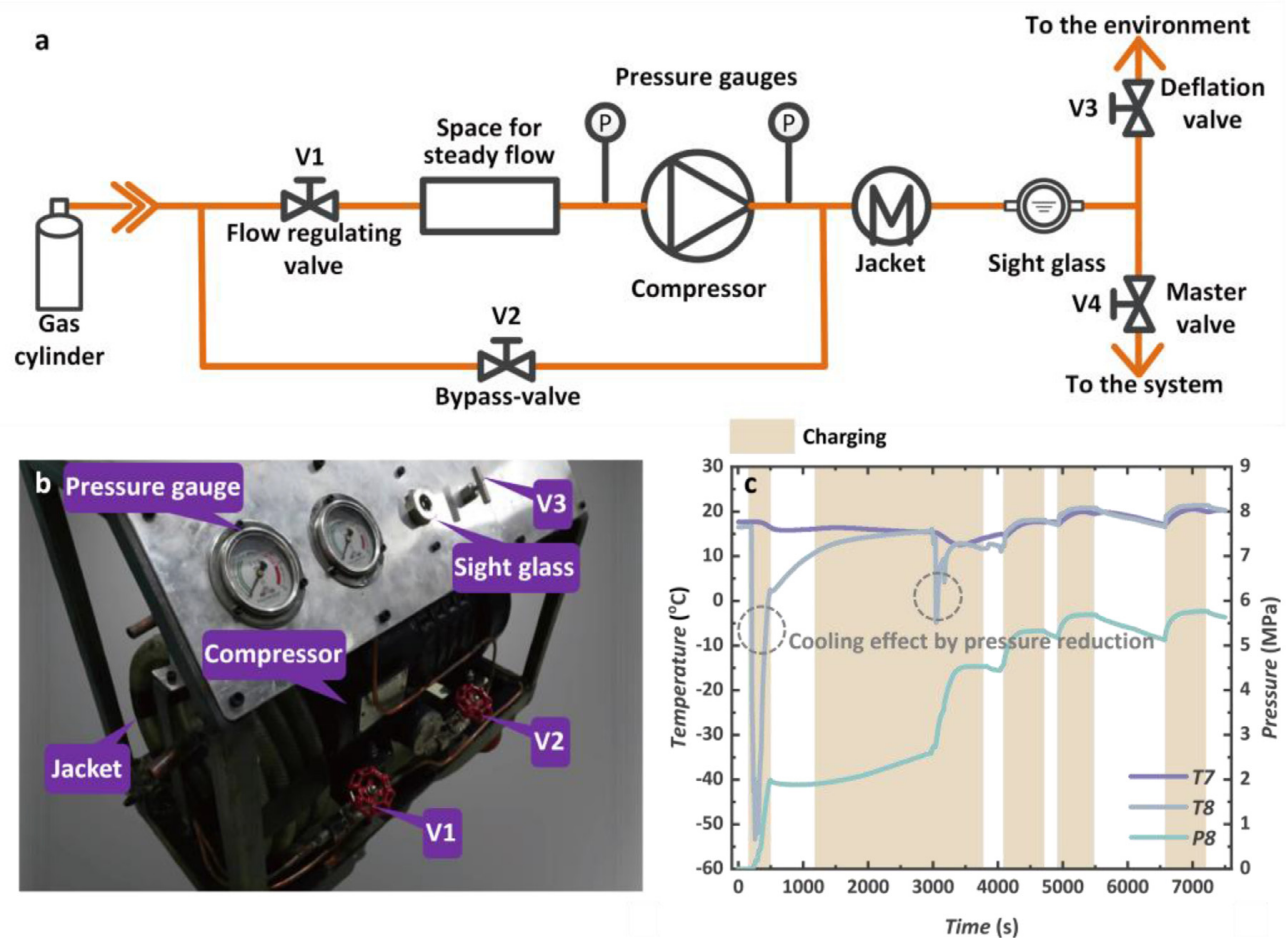
**CO_2_ filling system**. (a) Schematic diagram of the filling system. (b) Photo of the filling system. (c) Dynamic temperature and pressure during the charging process.

A portable filling system was constructed to charge the CO_2_ into the CTPC system. The schematic diagram and photo are shown in Fig. 3. The filling system includes a CO_2_ gas cylinder, compressor, water jacket, sight glass, and necessary valves with different functions. A typical filling process is shown in Fig. 3c demonstrating the dynamic response of the system pressure (P8) and temperature (T7 and T8). Brown regions represent the charging process. When the charging is started, the pressure is reduced quickly before and after the master valve V4. Hence, a large temperature drop of CO_2_ (T8) is observed and it becomes a low-pressure liquid state so that the minimum temperature reaches −50 °C. With the increase in the pressure difference between the compressor outlet and the CTPC system, a larger temperature drop will be found. And this is the reason why the minimum temperature only reaches −5 °C during the decompression of the second time. A more moderate trend is observed at the measure points away from the filling position such as T7, whose dynamic response is mainly determined by the system pressure.

### 2.3 Data acquisition system

**Fig. 4:**
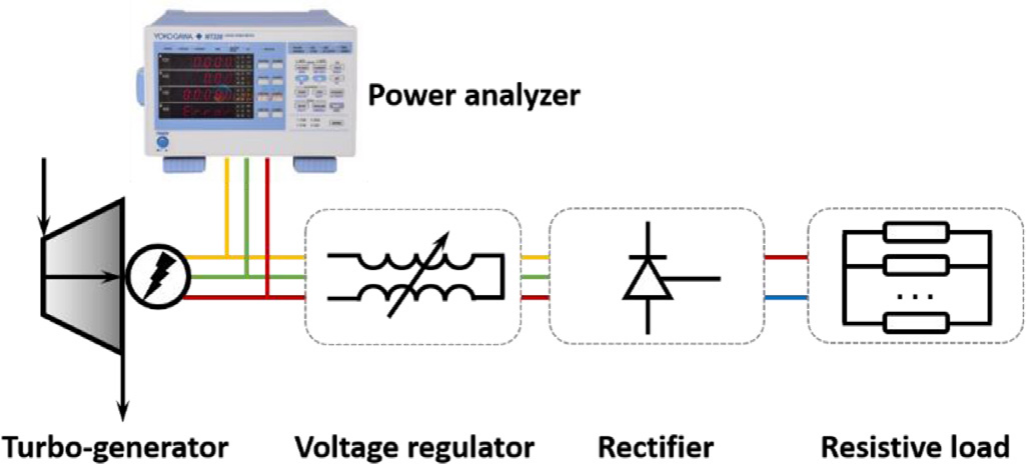
**Schematic diagram of the TG load system**. The voltage regulator was used to adjust the output voltage. The rectifier converted alternating current into direct current, and resistive loads were used to consume electricity.

For the engine control system, a standard measuring and controlling system was equipped to control the operation of the engine, and to measure crucial operation parameters including speed, torque, power and, fuel consumption rate. The measurement of the CTPC system consists of temperature, pressure, mass (volume) flow rates, rotational speeds, and electric power measurements. The measurement positions are shown in Fig. 2. The instrumentation specifications are presented in Table 3. There were 14 temperature sensors from T1 to T9 to measure the temperature of the working fluid at the inlet and outlet states of each component. Another 7 temperature sensors were used to measure the temperature of the engine exhaust gas (Te), engine coolant (Tc), and chilled water (Tch). Concerning the measurement of pressure, seven pressure transmitters were used to obtain the pressure of the working fluid, and another three pressure transmitters were arranged to monitor the pressure of the engine exhaust (Pe). There were 2 Coriolis flowmeters at each splitting branch to measure the mass flow rate of the working fluid. Another 3 flowmeters were constructed to detect the flow rate of engine exhaust gas, coolant, and chilled water. For the TG system, we developed an individual load system to monitor the TG operation and consume the electricity produced by the generator, the schematic diagram of which is shown in Fig. 4. For the resistive load, there are 8 resistances connected in parallel, and 8 kinds of different resistive loads can be achieved by the permutation. The specific values of the resistance are listed in Table 4. During the test at each engine condition and each pump speed, eight stages were conducted during the operation of the TG. The resistance values increased from 10.6 to 51.4 Ω; thus, a corresponding increase in the turbine rotational speeds would be achieved according to Ohm's law. Signals produced by such transducers are channeled through the PLC modules. A LabVIEW data acquisition program was developed to serve as the interface.

**Table 3:** **Specification of the measurement devices**.

Measured parameter	Physical principle	Range	Accuracy
Engine			

Speed	Dynamometer	0–3300 rpm	±1 rpm
Torque	Dynamometer	0–3055 N m	±0.1%
Fuel consumption rate	Coriolis	0–200 kg/h	±0.12%

Temperature			

Exhaust	Thermocouple Type K	−60–650 °C	±1%
CO_2_	Thermal resistance PT100	−200–600 °C	±0.15%
Water	Thermal resistance PT100	−200–600 °C	±0.15%

Pressure			

Exhaust	Differential pressure	0–0.5 MPa	±0.065%
CO_2_	Differential pressure	0–16 MPa	±0.065%

Flow rate			

CO_2_	Coriolis	0–0.8 kg/s	±0.2%
Exhaust	Hot-wire	0–1350 kg/h	±0.5%
Coolant	Turbine	1–10 m^3^/h	±0.5%
Chilled water	Turbine	1.5–15 m^3^/h	±1%

Turbo-generator			

AC power	Power analyzer	0–20 kW	±(MV × 0.1%+MR × 0.05%)
Current	Power analyzer	5–20,000 mA	±(MV × 0.1%+MR × 0.05%)
Voltage	Power analyzer	15–600 V	±(MV × 0.1%+MR × 0.05%)
Rotational speed	Photoelectric	0–45,000 rpm	±5%

**Table 4:** **The resistive load was connected during the test**.

Stage	360 Ω	180 Ω	180 Ω	72 Ω	60 Ω	60 Ω	60 Ω	60 Ω	Resistance value [Ω]
Ⅰ	●^a^	●	●	●	●	●	●	●	10.6
Ⅱ	●	●	●	●	●	●	●	○^b^	12.9
Ⅲ	●	●	●	●	●	●	○	○	16.4
Ⅳ	●	●	●	●	●	○	○	○	22.5
Ⅴ	●	○	●	●	●	○	○	○	25.7
Ⅵ	●	○	○	●	●	○	○	○	30.0
Ⅶ	●	●	○	○	●	○	○	○	40.0
Ⅷ	●	○	○	○	●	○	○	○	51.4

a ●: connected in parallel.

b ○: unconnected.

## 3 Results and discussion

**Fig. 5:**
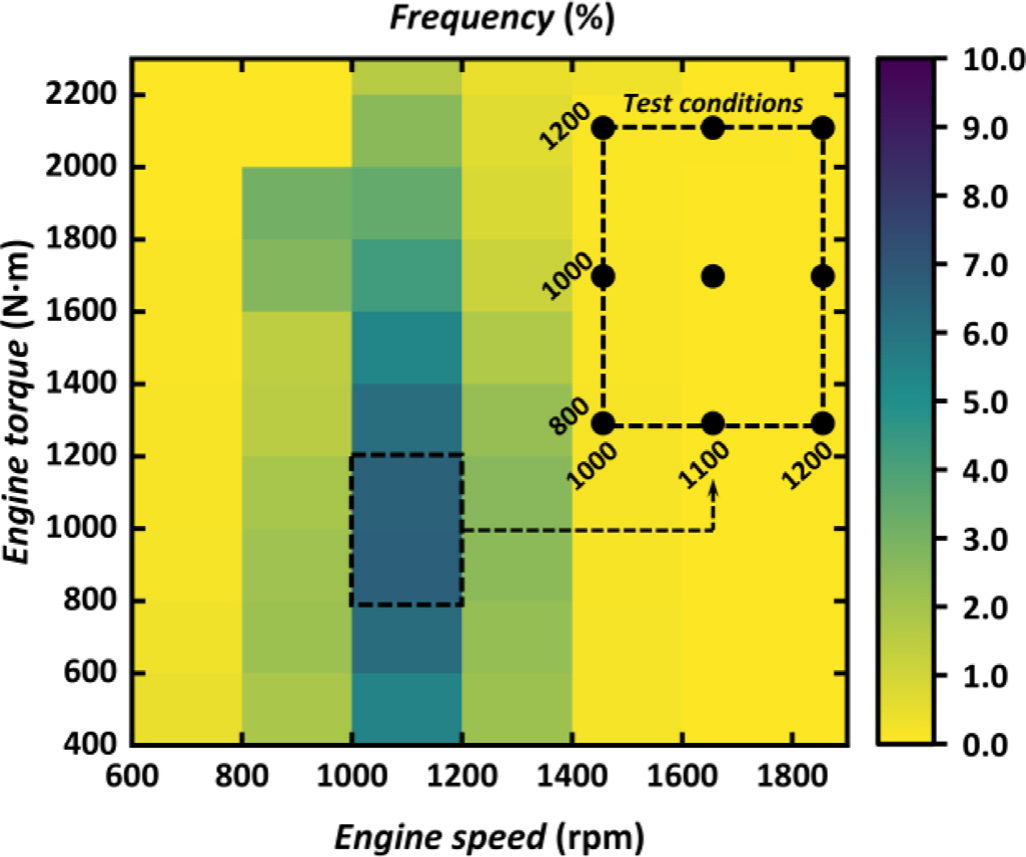
**Selected nine engine conditions based on-road data**.

**Fig. A1:**
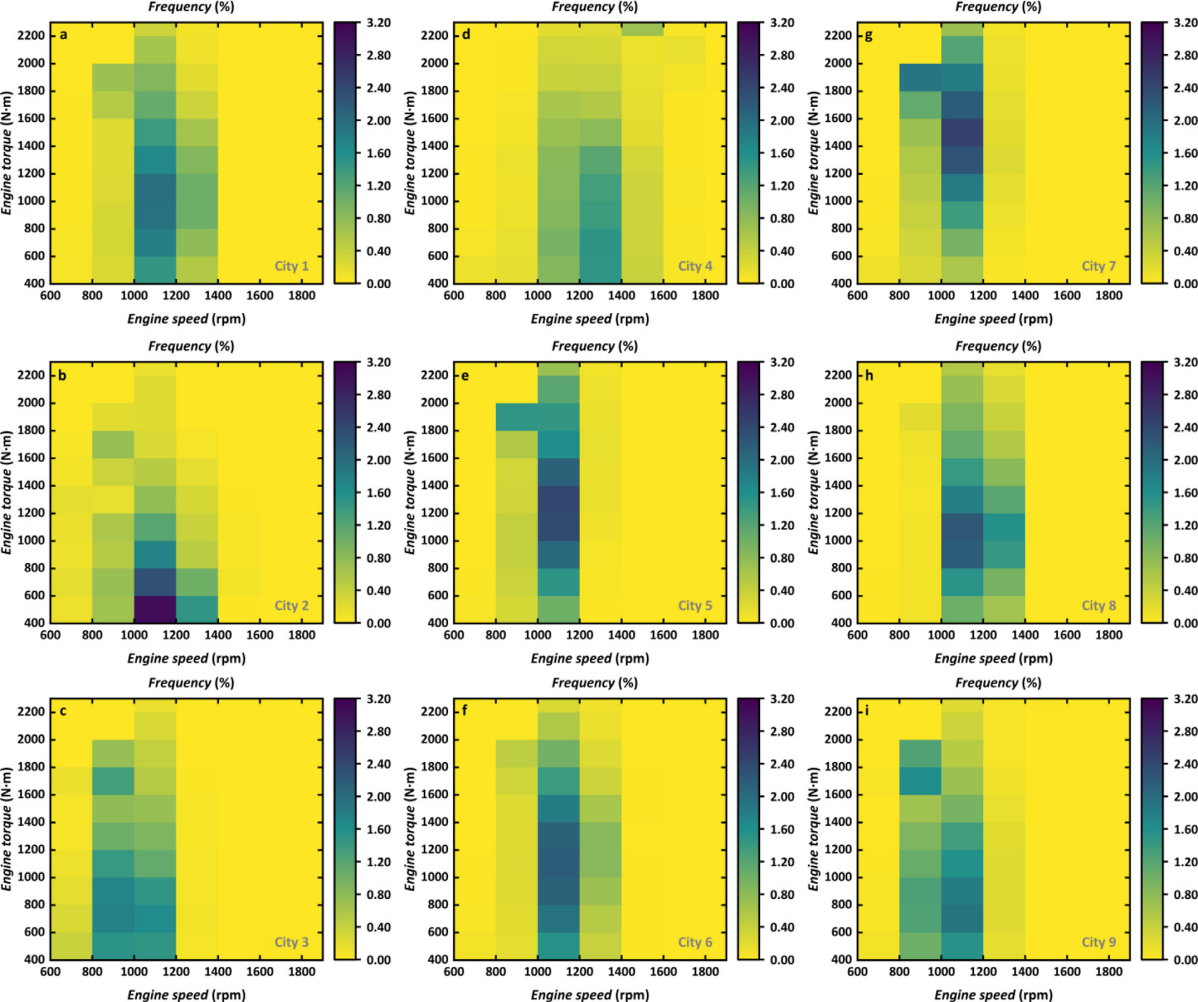
**On-road data from nine cities: time spent on each engine operation condition compared with the total operated hours**.

The input of the heat source is determined by the real on-road conditions of vehicles based on a large amount of on-road real-time data. Obtained in nine different cities, these data are measured from heavy-duty vehicles with the same engine being used and the average frequency of the engine operating conditions is evaluated, as shown in Fig. A1. By recording the time spent on each engine condition, a frequency map diagram could be summarized showing the ratio of time on each engine condition to the total hours operated. The summation of the total frequency reached 100%. Nine engine conditions were finally selected at which the engine operated the most. Based on the frequency of the engine speed and torque, these nine conditions are mainly concentrated on the region of medium speed, low and medium torque, shown in Fig. 5. Hence, the input of the heat source is provided by the engine operated at these nine common engine conditions covering the engine speed from 1000 rpm to 1200 rpm and engine torque from 800 N m to 1200 N m.

### 3.1 Transient characteristics

#### 3.1.1 Key components

**Fig. 6:**
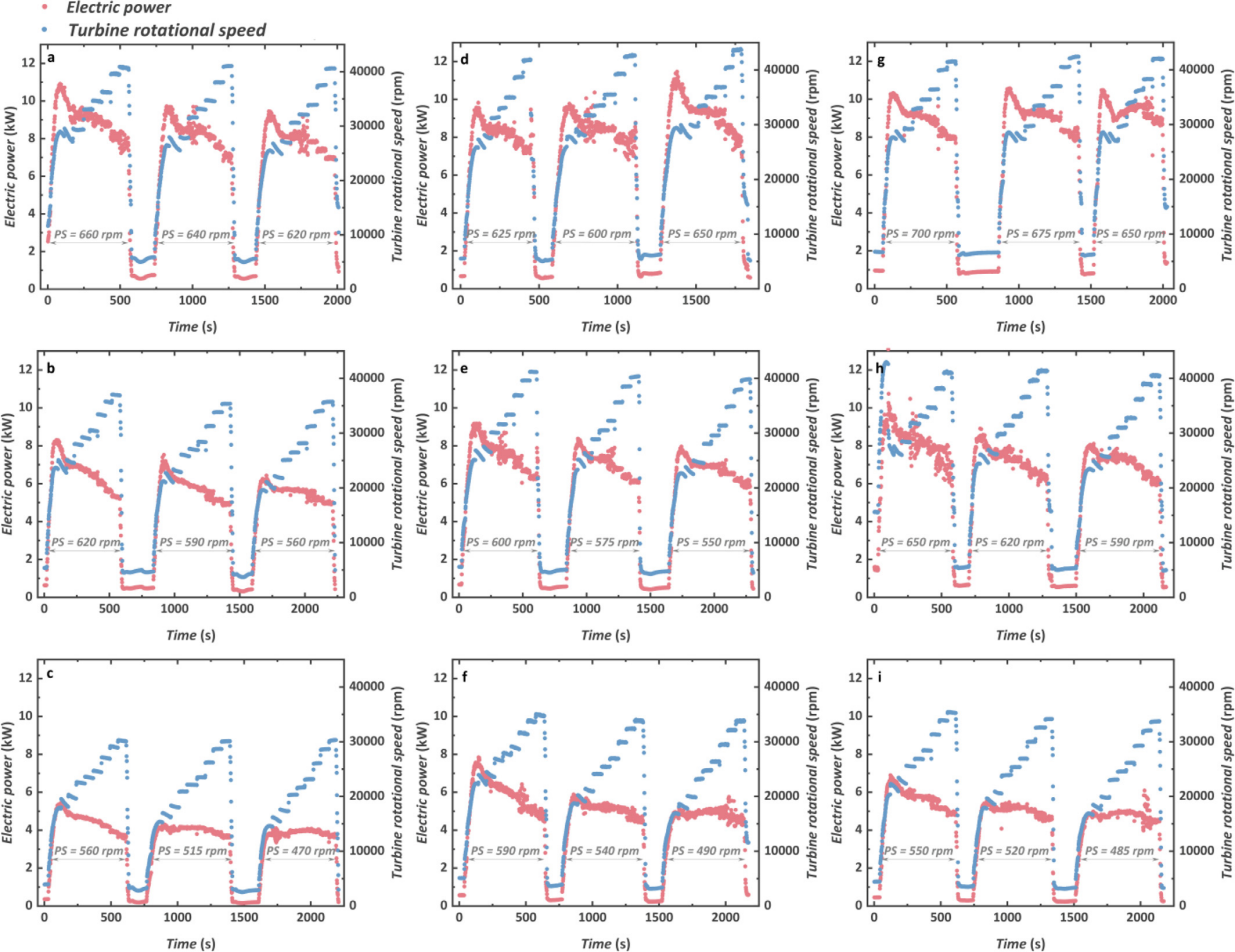
**Transient characteristics of the electric power (in red points) produced by TG and its rotational speed (in blue points)**. (a) 1000 rpm and 1200 N m. (b) 1000 rpm and 1000 N m. (c) 1000 rpm and 800 N m. (d) 1100 rpm and 1200 N m. (e) 1100 rpm and 1000 N m. (f) 1100 rpm and 800 N m. (g) 1200 rpm and 1200 N m. (h) 1200 rpm and 1000 N m. (i) 1200 rpm and 800 N m.

Dynamic responses of the electric power produced by the TG and its rotational speed at nine test engine conditions are shown in Fig. 6. The TG rotational speed and electric power were changed with various stages of resistive loads. For nine engine conditions, the highest electric power reaches 11.55 kW with the turbine rotational speed of 29,369 rpm at the engine condition of 1100 rpm, 1200 N m. With the increase in engine torque, the maximum electric power increases when the PS is kept the same. Taking the engine speed of 1200 rpm as an example, the maximum electric power of 7.85 kW can be obtained at 800 N·m, whereas the maximum one reaches 7.99 kW at 1000 N m when the PS is kept at 590 rpm. When the TG starts to operate, an overshoot phenomenon is observed in electric power. That is to say, the electric power increases sharply and then falls to some extent during the start-up stage of the TG, which is derived from the dynamic response of the turbine inlet pressure (explained in Fig. 9 in Section 3.1.2). For each engine condition, the electric power usually increases with the increase of the PS. The reason is that when the PS increases, the CO_2_ mass flow rate increases and also causes an increase in turbine inlet pressure. Although the turbine inlet temperature may decrease, the electric power increases because of a larger impact caused by the mass flow rate. Nevertheless, we believe that there must exist an optimal PS at each engine condition when the maximum electric power can be obtained.

Concerning the turbine rotational speed shown in blue dots, higher values are observed with the increase of both engine speed and torque, at which the CTPC system can absorb a larger quantity of waste heat. The TG rotational speed shows great relevancy to the variation of the engine condition although the PS is kept the same. For instance, when the PS operates at 620 rpm at engine conditions of 1000 rpm, 1200 N m and 1000 rpm, 1000 N m, respectively, the maximum turbine rotational speed declines from 40,648 rpm to 37,046 rpm with the same resistances. More waste heat could be recovered at a higher engine torque and the working fluid could be heated to a higher temperature. Hence, the turbine inlet temperature at 1200 N m is higher than that of 1000 N·m. Despite the mass flow rates being kept at the same level, a higher turbine inlet enthalpy could be achieved which improved the power capability with higher rotational speed and electric power. The maximum rotational speed reaches 43,872 rpm at the engine condition of 1100 rpm, 1200 N m with the PS of 650 rpm. The upper limit of the turbine rotational speed was designed to be 40,000 rpm, and when the maximum turbine rotational speed reached the maximum value, a higher value of PS was not tested considering the safety operation. Concerning each engine condition, an opposite trend is observed that the electric power decreases gradually when the turbine rotational speed increases. When the rotational speed is over 36,000 rpm, the electric power usually declines obviously due to the increase of the windage loss. It takes a very short period for the turbine rotational speed to become stable according to the dynamic response, which seems to be beneficial because the electric voltage and current are also stable.

**Fig. 7:**
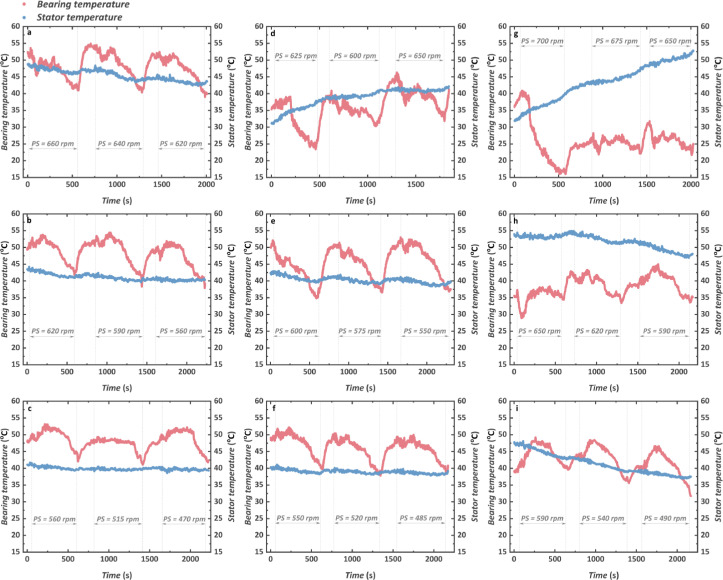
**Transient characteristics of key temperatures including the bearing temperature (in red points) and the stator temperature (in blue points)**. (a) 1000 rpm and 1200 N m. (b) 1000 rpm and 1000 N m. (c) 1000 rpm and 800 N m. (d) 1100 rpm and 1200 N m. (e) 1100 rpm and 1000 N m. (f) 1100 rpm and 800 N m. (g) 1200 rpm and 1200 N m. (h) 1200 rpm and 1000 N m. (i) 1200 rpm and 800 N m.

Facing high-speed operation and high-temperature working fluid, it is important to monitor the temperature of the key parts of the TG in case of some accidental damage caused by the unbearable thermal load. Hence, the transient process of the key parts’ temperature of the TG including the bearing temperature and stator temperature is shown in Fig. 7. On one hand, the bearing temperature shows a great correlation to the start-up of the turbine, and the temperature rise of 10−15 °C is observed after the running. Note that the bearing temperature does not increase when the turbine rotational speed increases and even a slight decrease can be observed at high rotational speeds. On the other hand, the TG stator temperature is more stable compared with the bearing temperature. With the increase of both engine speed and torque, the maximum stator temperature also increases slightly. Nevertheless, benefiting from the design of the resistant gas, the stator temperature is mainly affected by the resistant gas at the outlet of the preheater from the CTPC system and is controlled to less than 55 °C, which protects the safe operation of the high-speed generator and proves the effectiveness of the resistant gas design.

**Fig. 8:**
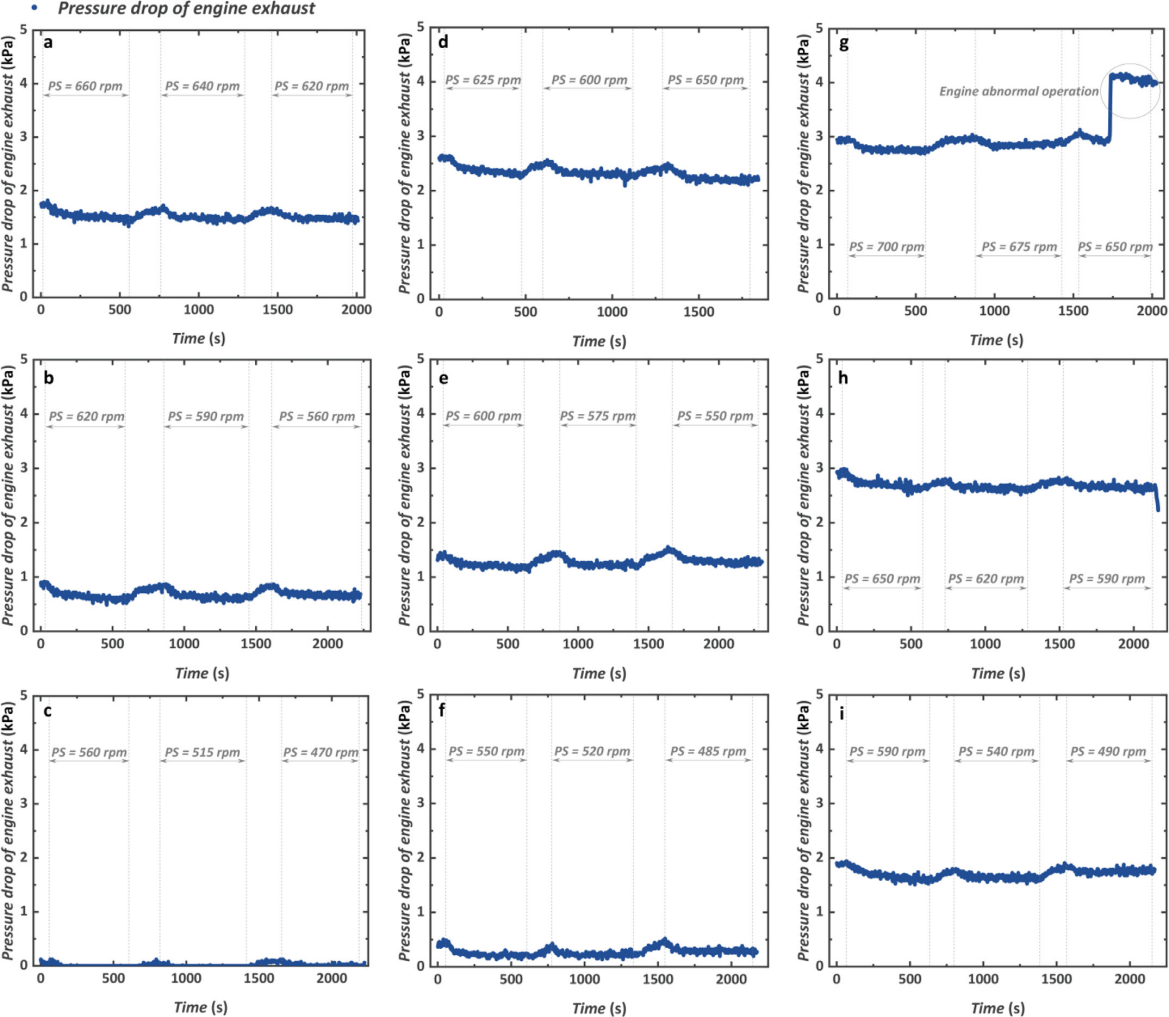
**Transient characteristics of the engine exhaust pressure drop**. (a) 1000 rpm and 1200 N m. (b) 1000 rpm and 1000 N m. (c) 1000 rpm and 800 N m. (d) 1100 rpm and 1200 N m. (e) 1100 rpm and 1000 N m. (f) 1100 rpm and 800 N m. (g) 1200 rpm and 1200 N m. (h) 1200 rpm and 1000 N m. (i) 1200 rpm and 800 N m.

Engine exhaust pressure drop has always been paid much attention in engine waste heat recovery because it affects the original engine to some degree. The dynamic response of the total exhaust pressure drop considering both the HTGH and LTGH at nine test conditions is shown in Fig. 8. Overall, the engine condition has a great impact on the pressure drop. When the engine speed is kept at 1100 rpm, the average pressure drop increases from 0.27 kPa to 1.28 kPa and 2.33 kPa with the increase of the engine torque from 800 N m to 1000 N m and 1200 N m. When the engine torque is kept at 1000 N m, the average pressure drop also increases from 0.68 kPa to 1.28 kPa and 2.68 kPa. That is to say, the exhaust pressure drop is not only affected by the mass flow rate of the engine exhaust, which is mainly determined by the engine speed, but also affected by the heat transfer temperature, which contributes to different exhaust viscosity. When the engine is operated at the maximum condition of 1200 rpm and 1200 N m with the value of PS being 650 rpm, the mass flow rate of the engine exhaust gas increases suddenly because of the abnormal operation of the engine control system. And the temperature of the engine exhaust decreases accordingly. Nevertheless, the pressure drop of the engine exhaust increases accordingly which is largely affected by the exhaust mass flow rate. In addition, the different pump rotational speed produces limited influence on the exhaust pressure drop.

#### 3.1.2 CTPC system

**Fig. 9:**
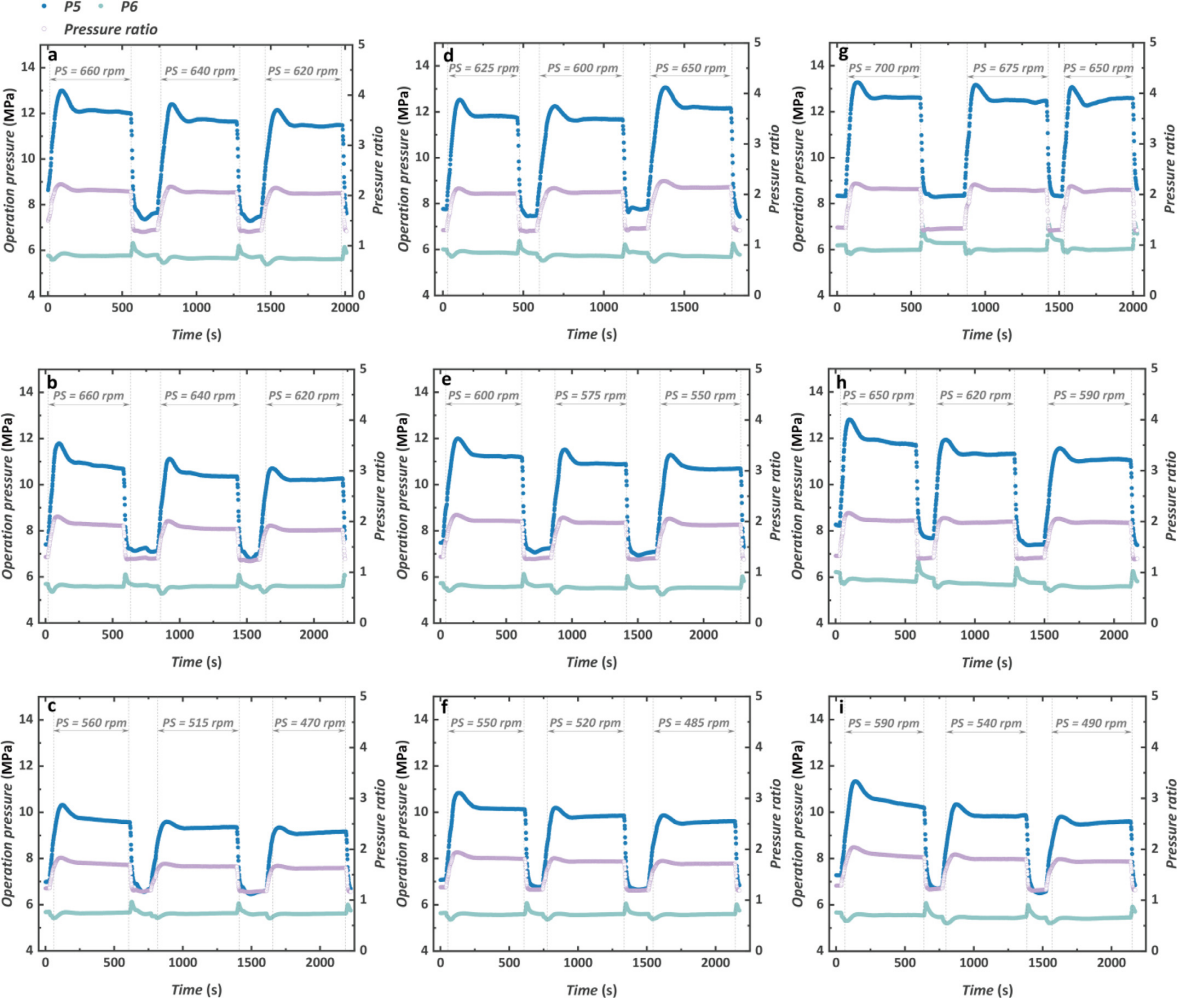
**Transient characteristics of the pressure of the working fluid**. (a) 1000 rpm and 1200 N m. (b) 1000 rpm and 1000 N m. (c) 1000 rpm and 800 N m. (d) 1100 rpm and 1200 N m. (e) 1100 rpm and 1000 N m. (f) 1100 rpm and 800 N m. (g) 1200 rpm and 1200 N m. (h) 1200 rpm and 1000 N m. (i) 1200 rpm and 800 N m.

The system operating pressure and pressure ratio are shown in Fig. 9. For nine test conditions, the high-pressure side turbine inlet pressure P5 increases with the increase of engine speed and torque. This is mainly determined by two reasons. On one hand, the increased mass flow rate of the CO_2_ in the system will lead to higher operating pressure. On the other hand, when a larger amount of waste heat is inputted into the system, the temperature of the working fluid will also increase and then affects the high pressure correspondingly. The maximum P5 reaches 13.3 MPa when the PS reaches 700 rpm at the engine condition of 1200 rpm, 1200 N m. The low-pressure side P6 is mainly determined by the condensation temperature. Hence, the value of P6 is without big changes during the stable operation of the TG and shows no relevance to the variation of the pump rotational speed.

The phenomenon of the overshoot in both P5 and P6 is observed again. Opposite tendencies in P5 and P6 are found when the turbine starts to operate. The P6 firstly decreases and then increases to a stable value. This is because when the turbine starts to rotate after being switched from the throttle valve, it takes a period for the rotational speed to increase to a stable state. The limited rotational speed is restricting the flow of the CO_2_ through the blade and causes a decrease in the turbine outlet pressure P6. When the turbine rotational speed increases further, both the pressure of P5 and P6 tend to be stable. For the engine condition with higher speed and torque, the overshoot in P5 is more apparent. The change in the pressure ratio is mainly determined by the P5 due to a relatively invariable value of P6. Hence, the pressure ratio also increases with the increase of the engine conditions. The variation of the pressure ratio for nine engine conditions is between 1.68 and 2.22. When the engine condition is kept the same, the increased PS will contribute to an increased pressure ratio.

**Fig. 10:**
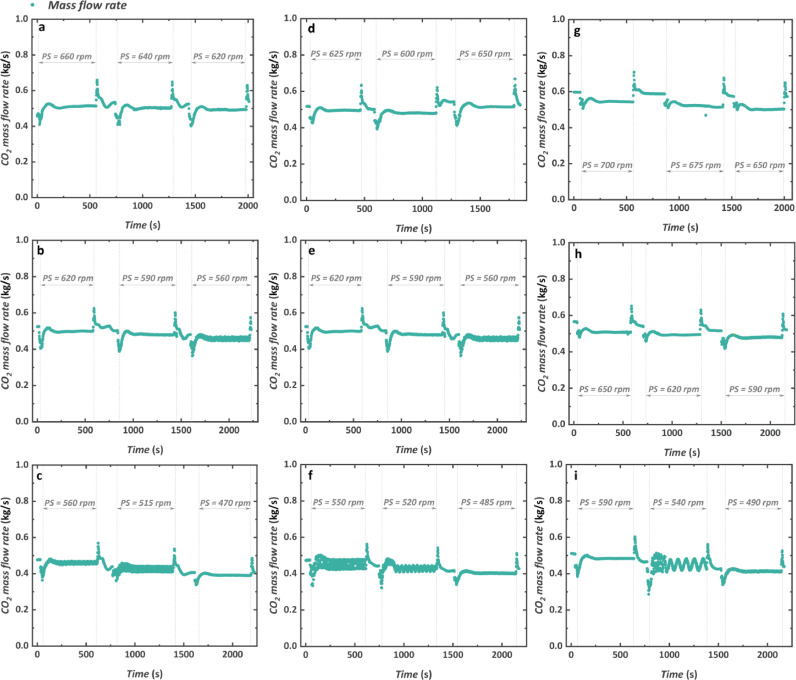
**Transient characteristics of the mass flow rate of the working fluid**. (a) 1000 rpm and 1200 N m. (b) 1000 rpm and 1000 N m. (c) 1000 rpm and 800 N m. (d) 1100 rpm and 1200 N m. (e) 1100 rpm and 1000 N m. (f) 1100 rpm and 800 N m. (g) 1200 rpm and 1200 N m. (h) 1200 rpm and 1000 N m. (i) 1200 rpm and 800 N m.

The dynamic response of the mass flow rate of the working fluid is shown in Fig. 10. The value of the mass flow rate in the CTPC system is mainly determined by the PS which is different from the situation in the supercritical CO_2_ Brayton cycle where the system pressure ratio affects the operation of the compressor and then determines the mass flow rate. To some extent, the control of the CTPC is not that complicated compared with that of the supercritical CO_2_ Brayton cycle. It can be found that there are two interesting phenomena about the transient characteristics of the mass flow rate. One is the overshoot phenomenon when the TG starts to operate or stops operating. The reason is that with the variation of the system pressure, shown in Fig. 9, the density at the pump inlet also changes. Specifically, with a decrease in the operating pressure, the density of CO_2_ decreases when the temperature is kept constant. When the CO_2_ pump operates at a constant rotational speed, the CO_2_ mass flow rate is primarily determined by its inlet density because the cylinder volume remains unchanged in the positive displacement pump.

When the operation of the turbine becomes stable, the mass flow rate nearly keeps unchanged except for some specific pump rotational speeds from 515 rpm to 560 rpm where some fluctuation is observed, which is supposed to be the second interesting phenomenon. The fluctuation should not be attributed to the vibration of the rotating TG in a certain mass flow rate region. We think it more reasonable that some specific pump rotational speeds will cause the vibration of the experimental rig, and consequently produce some impacts on the measurement of the Coriolis mass flowmeter so that the oscillation in the measured CO_2_ mass flow rate is observed. Nevertheless, the real mass flow rate in the system is supposed to be stable as well exactly as other pump rotational speeds.

**Fig. 11:**
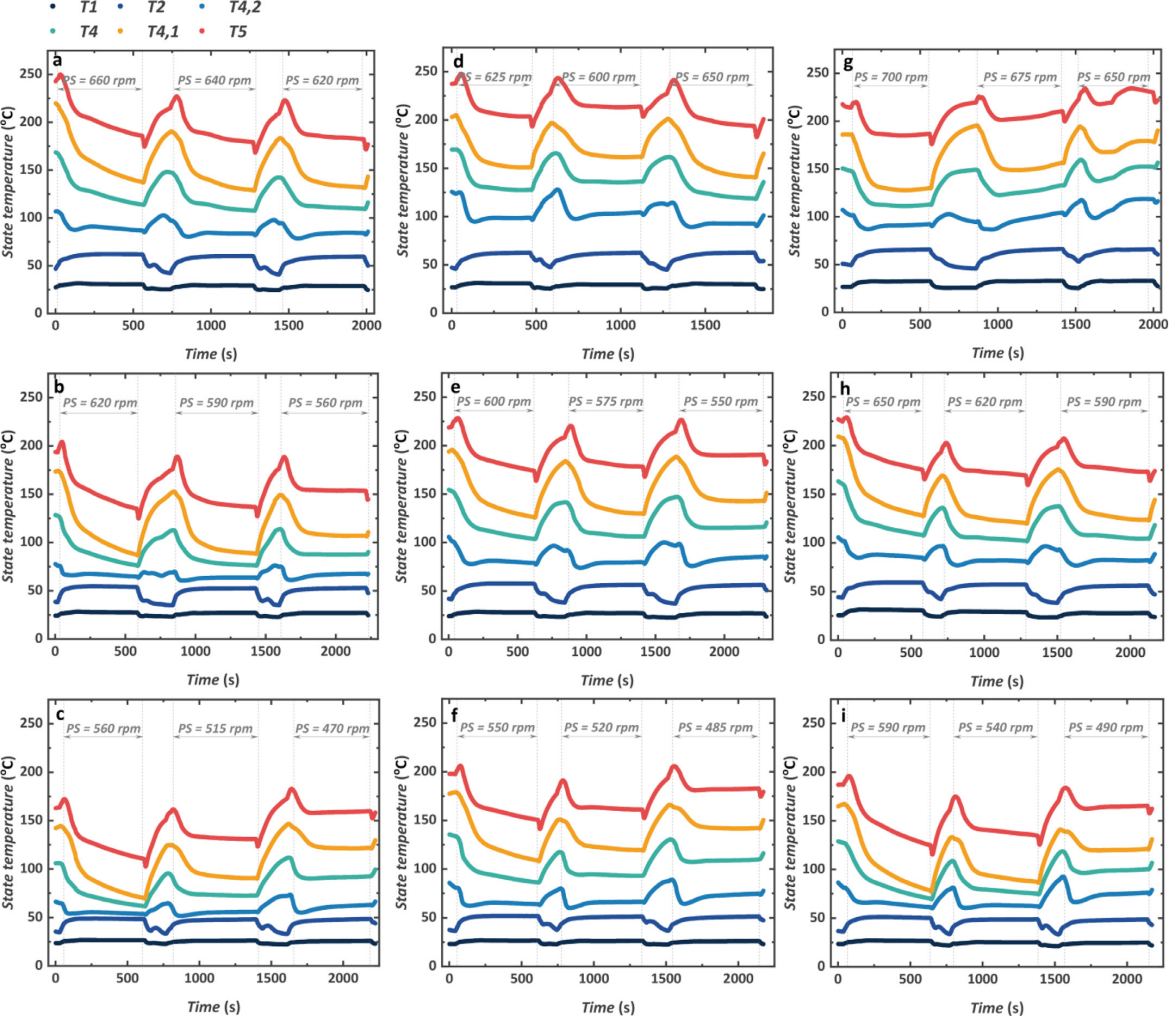
**Transient characteristics of the temperature of the high-pressure side working fluid**. (a) 1000 rpm and 1200 N m. (b) 1000 rpm and 1000 N m. (c) 1000 rpm and 800 N m. (d) 1100 rpm and 1200 N m. (e) 1100 rpm and 1000 N m. (f) 1100 rpm and 800 N m. (g) 1200 rpm and 1200 N m. (h) 1200 rpm and 1000 N m. (i) 1200 rpm and 800 N m.

**Fig. 12:**
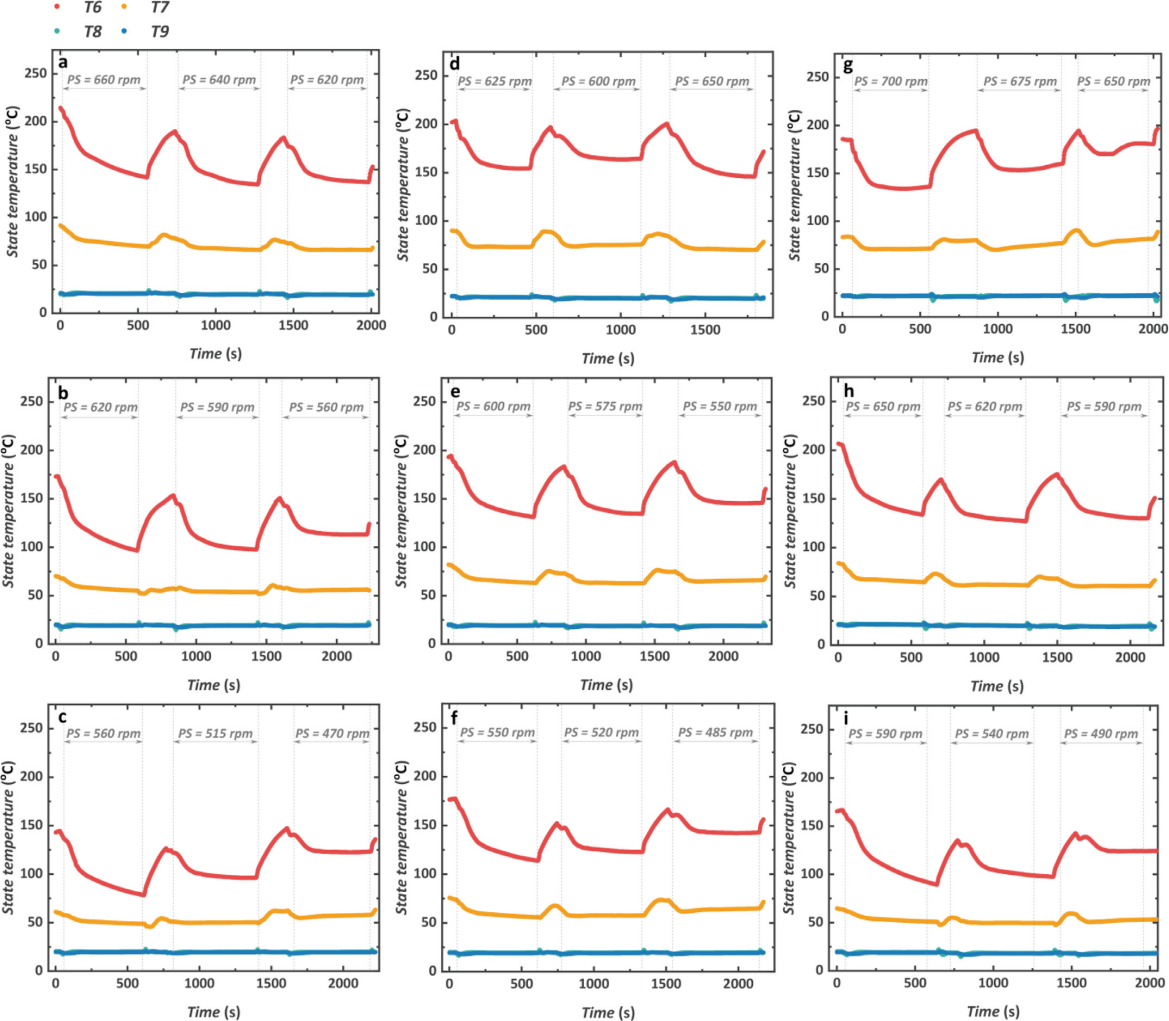
**Transient characteristics of the temperature of the low-pressure side working fluid**. (a) 1000 rpm and 1200 N m. (b) 1000 rpm and 1000 N m. (c) 1000 rpm and 800 N m. (d) 1100 rpm and 1200 N m. (e) 1100 rpm and 1000 N m. (f) 1100 rpm and 800 N m. (g) 1200 rpm and 1200 N m. (h) 1200 rpm and 1000 N m. (i) 1200 rpm and 800 N m.

The dynamic process of the temperature measurement of each state is shown in Fig. 11, Fig. 12, demonstrating the temperature of the high-pressure side working fluid from T1 to T5 and low-pressure side working fluid from T6 to T9, respectively. Concerning the nine engine conditions, the highest system temperature increases with the increase of the engine conditions changing from less than 200 °C to more than 250 °C. A decrease in turbine inlet temperature T5 is observed after the switch from the throttle valve when the turbine starts to operate. That is to say, the decompression process of the throttle valve is different from the practical operation of the turbine. The real expansion process leads to a decrease in the turbine outlet temperature T6 which also produces an impact on the heat transfer in the recuperator. Hence, the state temperature of T4 also decreases, which reflects the mixing process of two splitting branches. Both the temperature of T4 and T5 increase back to the temperature when the throttle valve is activated after the turbine stops running. Noted that the value of T4,1 and T4,2 also has a similar rising trend when the turbine stops running due to the increase in T6. Nevertheless, the value of T4,1 saw a more rapid increase compared with T4,2. In addition to a different thermodynamic process between the turbine and the throttle valve, the reason is also attributed to the splitting design of the power cycle. The mass flow rate through each splitting branch is determined by the pressure drop in the recuperator and LTGH. To achieve the same pressure drop, the mass flow rate of CO_2_ is different from each other in both heat exchangers, which produces different impacts on the heat transfer process.

The dynamic response of the preheater outlet temperature T2 shows a contrary trend that a moderate decrease is observed when the turbine stops running. This is because the CO_2_ mass flow rate increases for an instant when the turbine stops running so the preheater outlet temperature decreases when the heat transfer in the preheater is kept the same. When the CO_2_ mass flow rate decreases gradually, the pace of the decline of T2 is slowed until the next time when the turbine starts to run.

For nine engine conditions, both T6 and T7 increase with the increase of the engine speed and torque. Nevertheless, the value of T8 and T9 shows little relevance to the change in engine conditions because they are determined by the condensation process. The value of T6 also decreases when the turbine starts to operate. This is mainly derived from the decreased T5 shown in Fig. 11. The temperature difference before and after the turbine is within the range of 30–50 °C. The recuperator outlet temperature T7 fluctuates less than that of T6. And the value of both T8 and T9 keeps nearly unchanged under a constant condensation condition.

### 3.2 Steady performance

#### 3.2.1 Key components

**Fig. 13:**
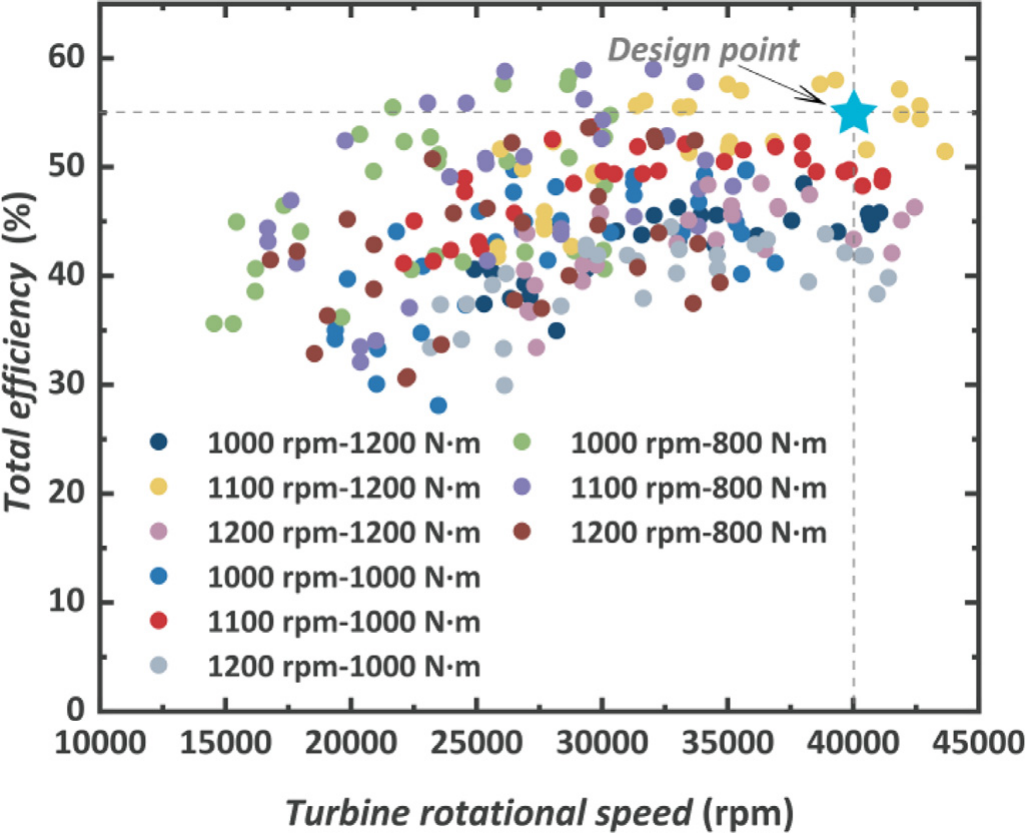
**Performance map of the TG at various engine conditions when the total efficiency is as a function of the turbine rotational speed**.

The total efficiency of the TG is shown in Fig. 13 demonstrating the product of turbine isentropic efficiency and electricity-generating efficiency. For all the steady points of nine engine conditions, because the maximum total efficiency and the maximum electric power are not obtained at the same condition, hence, the maximum TG total efficiency (58.92% under 29,251 rpm) is not obtained at engine conditions with higher speed and torque, at which the TG produces higher electric power. For each test engine condition, there is an optimal turbine rotational speed to achieve the maximum total efficiency. Also, the optimal rotational speed shifts to a relatively low value when the engine speed and torque are relatively low. Although the shaft work produced by the turbine cannot be measured, the maximum turbine isentropic efficiency is assessed to be around 65% considering the generating efficiency of 90% to 95%.

**Fig. 14:**
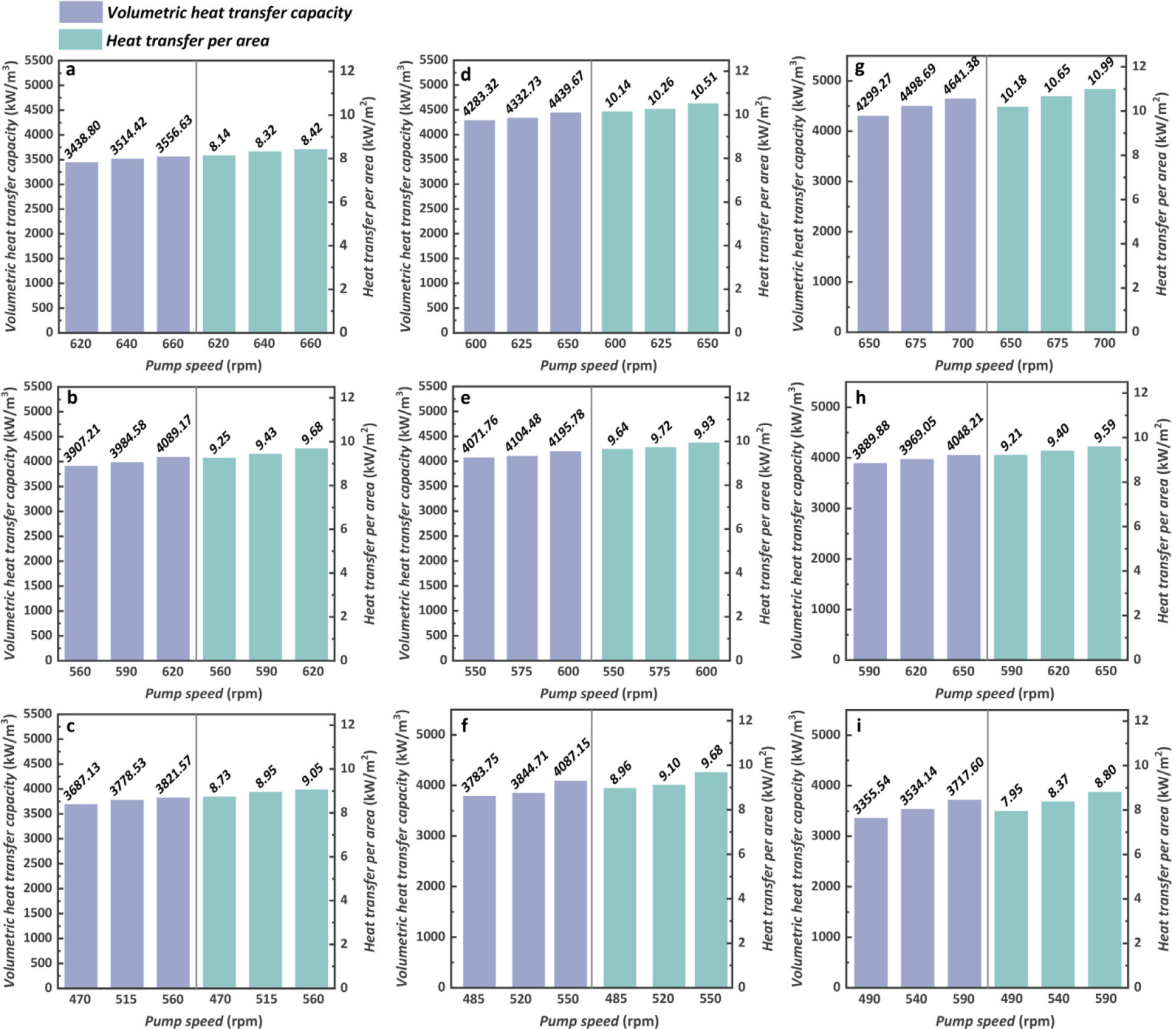
**Heat transfer performance and compactness assessment of the exhaust-sCO_2_ PCHE (HTGH)**. (a) 1000 rpm and 1200 N m. (b) 1000 rpm and 1000 N m. (c) 1000 rpm and 800 N m. (d) 1100 rpm and 1200 N m. (e) 1100 rpm and 1000 N m. (f) 1100 rpm and 800 N m. (g) 1200 rpm and 1200 N m. (h) 1200 rpm and 1000 N m. (i) 1200 rpm and 800 N m.

To figure out the heat transfer of sCO_2_-exhaust PCHE and its compactness, two indicators including the volumetric heat transfer capacity (calculated by heat absorption divided by its volume) and heat transfer per area (calculated by heat absorption divided by its heat transfer area) are shown in Fig. 14 taking the HTGH into account. Both the volumetric heat transfer capacity and heat transfer per area show great relevance to the engine conditions that a higher engine speed or torque will lead to an increase in both indicators. The maximum values reach 4641.38 kW/m^3^ and 10.99 kW/m^2^ with a heat absorption rate of 65.61 kW when the engine operates at 1200 rpm, 1200 N m. Although the pump speed is kept the same, the difference in engine conditions also produces an impact on these two indicators. For a constant engine condition, both the volumetric heat transfer capacity and heat transfer per area increase with the increase in pump speed.

#### 3.2.2 CTPC system

**Fig. 15:**
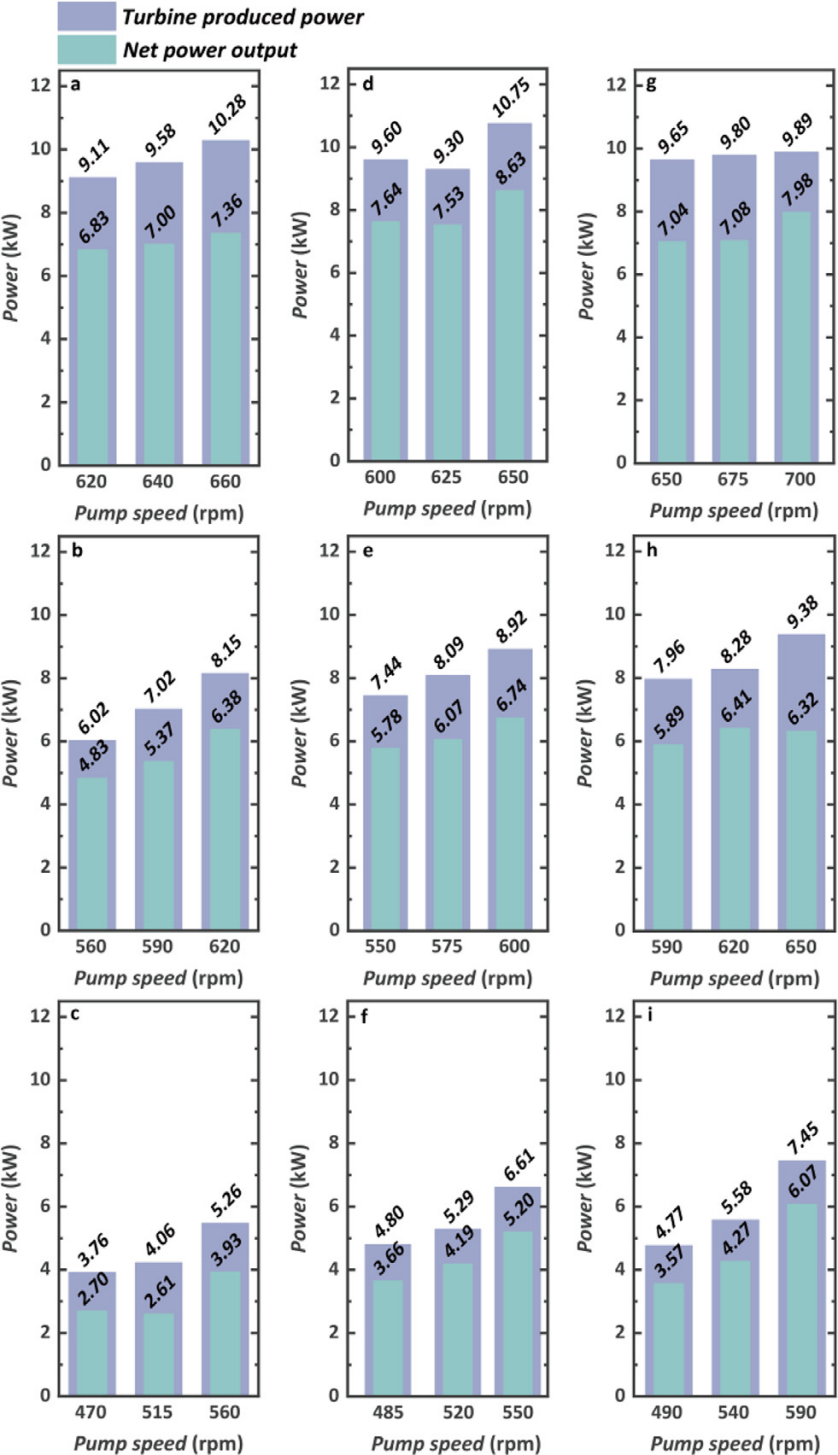
**Thermodynamic performance evaluation considering the produced and consumed power**. (a) 1000 rpm and 1200 N m. (b) 1000 rpm and 1000 N m. (c) 1000 rpm and 800 N m. (d) 1100 rpm and 1200 N m. (e) 1100 rpm and 1000 N m. (f) 1100 rpm and 800 N m. (g) 1200 rpm and 1200 N m. (h) 1200 rpm and 1000 N m. (i) 1200 rpm and 800 N m.

The produced power by TG, consumed power by the pump, and net power output are shown in Fig. 15. Both the engine condition and pump rotational speed have a great effect on system power capability. Taking the engine speed of 1200 rpm as an example, when the pump rotational speed is kept at 650 rpm, the net power output reaches 7.04 kW and 6.32 kW at engine conditions of 1200 N m and 1000 N m, respectively. When the engine condition is kept the same, all the produced power by TG, consumed power by the pump, and net power output increase with the pump's rotational speed in most cases. Nevertheless, although the optimal pump rotational speed has not been found according to the experimental results, we think that there is an optimal pump rotational speed where the maximum net power output can be obtained and the net power output will decrease with the further increase in pump rotational speed because the pump consumed power will increase apparently if the pump rotational speed increases further, and the increase in turbine produced power will not cover the increase in pump consumed power. In another word, there is still much room for the system's power capability to improve.

**Fig. 16:**
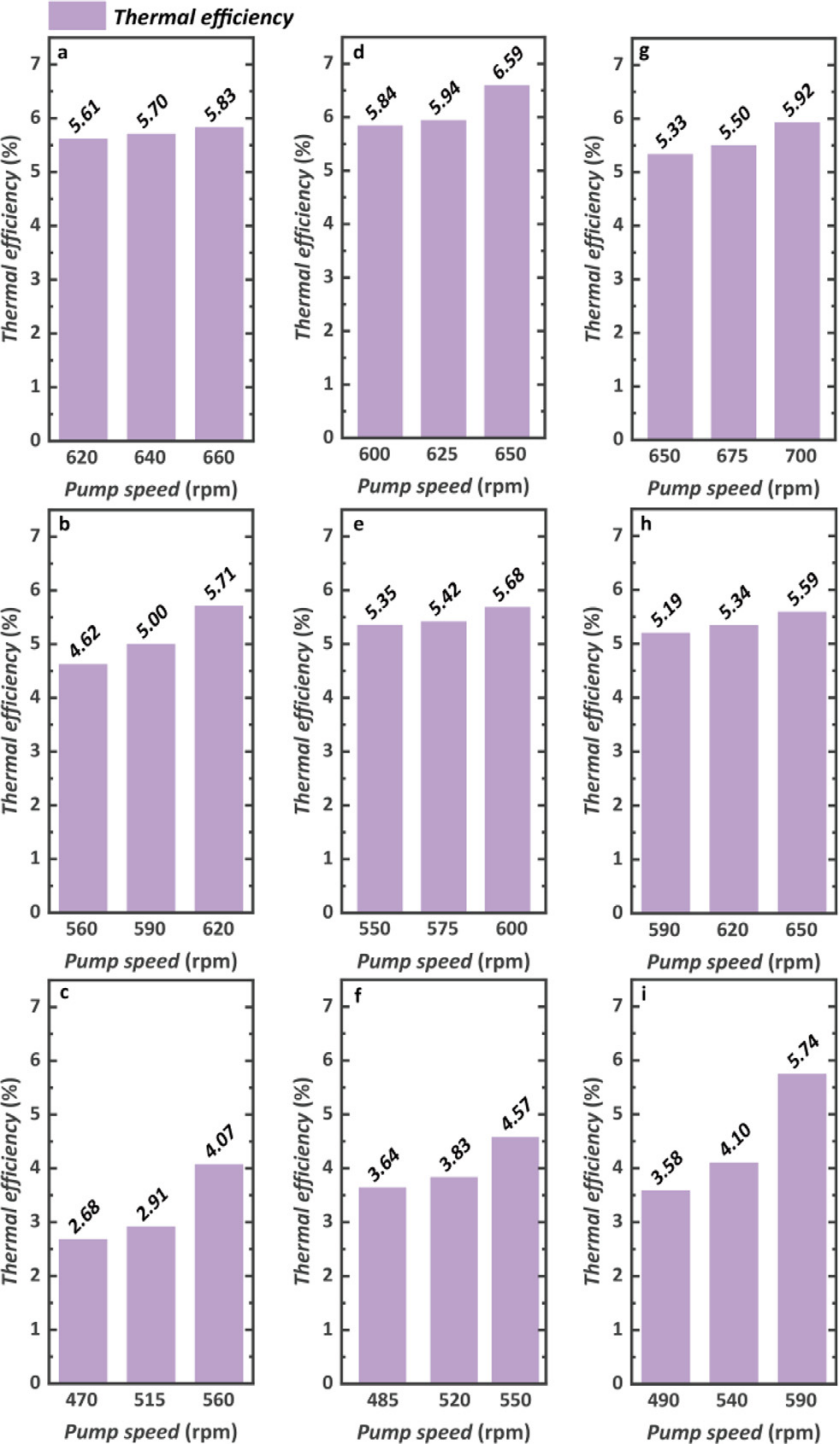
**Thermodynamic performance evaluation considering the thermal efficiency**. (a) 1000 rpm and 1200 N m. (b) 1000 rpm and 1000 N m. (c) 1000 rpm and 800 N m. (d) 1100 rpm and 1200 N m. (e) 1100 rpm and 1000 N m. (f) 1100 rpm and 800 N m. (g) 1200 rpm and 1200 N m. (h) 1200 rpm and 1000 N m. (i) 1200 rpm and 800 N m.

The system thermal efficiency is shown in Fig. 16. This value is determined by the net power output and heat absorption rate. For nine engine conditions, the maximum thermal efficiency reaches 6.59% at the engine condition of 1100 rpm, 1200 N m. Because the higher engine speed and torque not only leads to a higher net power output but also promotes heat absorption, the thermal efficiency may decline with higher net power output. For instance, when the engine operates at 1000 rpm, 1000 N m, and 1200 rpm, 1000 N m with the same pump rotational speed of 620 rpm, the thermal efficiency decrease from 5.71% to 5.34% although the net power output increases from 6.38 kW to 6.41 kW. Hence, the higher thermal efficiency does not mean a higher net power output at the same time. It should be noted that the engine condition has a profound impact on thermal efficiency. For the engine speed of 1000 rpm, the maximum thermal efficiency for 800 N m is decreased by 30% compared with that of 1200 N m. Hence, it is necessary for the research not only to consider the performances at the design point but also to take the engine operation profile into account.

### 3.3 Energy saving potential

**Fig. 17:**
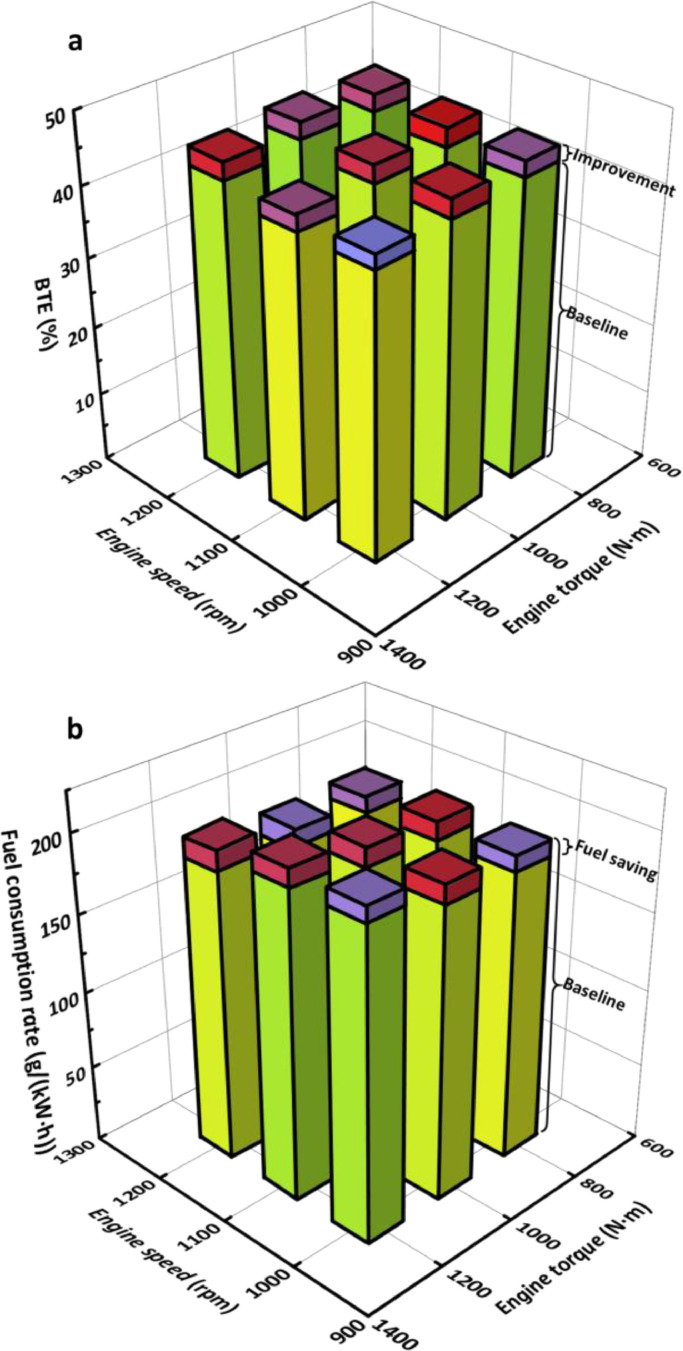
**Energy conservation potential compared with the baseline of the original engine**. (a) BTE improvement. (b) Fuel saving.

Integrated with the CTPC engine waste heat recovery system, the energy saving potential including the engine brake thermal efficiency (BTE) improvement and fuel consumption reduction for nine tested engine conditions are shown in Fig. 17. The energy saving potential for each engine condition is different. The maximum BTE is increased by 6.3% relatively compared with the original BTE at the engine condition of 1100 rpm, 1200 N m from 42.8% to 45.5%, whereas the maximum BTE with the CTPC system reaches 46.2% when the engine operates at 1200 rpm, 1000 N m. Concerning the fuel-saving potential, the fuel consumption rate decreases from 196.9 g/(kW h) to 184.6 g/(kW h) at the engine condition of 1100 rpm, 1200 N m. For other tested engine conditions, the minimum fuel saving is higher than 9.8 g/(kW h) when the engine condition is 1000 rpm, 800 N m, which is also supposed to be a fuel-efficient technology especially when it is encountered in low engine conditions.

## 4 Conclusions and future works

In this research, a detailed experimental investigation of the engine-CTPC waste heat recovery system is carried out evaluating the performance of both the key components and CTPC system in the transient and steady-state taking the most frequent nine engine conditions based on road load data into account. Key components including a 10 kW-scale turbine integrated with a generator and two exhaust-sCO_2_ PCHEs for HTGH and LTGH are developed. In addition, a filling system is manufactured to charge the working fluid into the CTPC system.

As far as the key component of TG is concerned, the design of the resistant gas has been proven to be an efficient means of dealing with the sealing and cooling problem in the sCO_2_ turbine. The maximum electric power produced by TG reaches 11.55 kW at the rotational speed of 29,369 rpm. Both the engine conditions and pump rotational speeds have a great impact on electric power and total efficiency. The maximum TG total efficiency reaches 58.92% and the isentropic efficiency is assessed to be around 65% considering the generating efficiency. Nevertheless, when the TG operates at conditions away from the design point, an obvious decrease in total efficiency is observed. Hence, it is also important to conduct specific optimization under off-design conditions. For different engine conditions, there is an optimal turbine rotational speed to obtain the maximum total efficiency. An interesting phenomenon of the overshoot in the electric power when the TG starts to operate is observed, which is attributed to the fluctuation in the system pressure. Hence, it is important to further explore the transient characteristics during the start-up and shutdown process of TG.

The developed exhaust-sCO_2_ PCHE is proved to be an effective and compact heat exchanger in engine exhaust waste heat recovery. On an efficient heat transfer base, the total exhaust pressure drop is less than around 4 kPa when nine engine conditions are considered according to the dynamic test results making sure of reducing the impact on the original engine as much as possible. The engine conditions affect the exhaust pressure drop mainly whereas limited impact is found caused by various pump rotational speeds. And the exhaust pressure drop does not increase apparently although we have tested the developed PCHE for more than one year. Nevertheless, we still think it necessary to conduct special endurance tests to validate its performances such as high-temperature resistance and low pressure drop. Also, the engine condition affects the heat transfer. The maximum volumetric heat transfer capacity and heat transfer per area reach 4641.38 kW/m^3^ and 10.99 kW/m^2^, respectively, showing great potential in miniaturization.

Different dynamic responses to the change in engine conditions and pump rotational speeds of the mass flow rate, temperature, and pressure of the CTPC system are observed. These three operating parameters also affect each other. Usually, the increase in engine conditions contributes to higher system turbine inlet pressure and temperature, whereas the impact on the turbine outlet pressure is inconspicuous. The system mass flow rate is mainly determined by the pump's rotational speed and affects the system temperature and pressure indirectly. The oscillation in CO_2_ mass flow rate when the pump rotational speed is between 515 rpm and 560 rpm is observed and it is speculated that the specific pump rotational speed causes the vibration of the test rig and affects the measurement of the Coriolis mass flowmeter.

Integrated test results revealed that the maximum thermal efficiency reaches 6.59% with a net power output of 8.63 kW. Concerning the energy-saving potential, different degrees in promoting the engine brake thermal efficiency and reducing the fuel consumption rate are achieved at all the tested engine conditions. The BTE could be significantly improved by 6.3% relatively and the fuel consumption rate could be reduced from 196.9 g/(kW h) to 184.6 g/(kW h). There is still an energy-saving effect even at low engine speed and torque (Appendix A).

There are two potential methods to improve the system performance further based on the current test rig. On one hand, it is necessary to figure out the optimal pump rotational speed at each engine condition to acquire higher net power output as illustrated before. On the other hand, the splitting design of the CTPC system makes it possible to control the mass flow rate of the CO_2_ through each splitting branch actively. Thus, a higher mixing temperature T4 and a higher turbine inlet temperature T5 can be achieved consequently when the total mass flow rate and turbine inlet pressure are kept the same by changing the opening of the electric valves in each branch, which results in an improvement in enthalpy difference of the turbine and net power output. In addition, the nine high-frequent engine conditions need to be expanded further to the total engine operation profile.

**Table:** 

**Nomenclature**	
*Symbols, abbreviations, and subscripts*	
BTE	brake thermal efficiency
c	engine coolant
ch	chiller water
CTPC	CO_2_ transcritical power cycle
HTGH	high-temperature gas heater
in	inlet
LTGH	low-temperature gas heater
MF	mass flow rate [kg/s]
mid	middle
MR	measurement range
MV	measurement value
out	outlet
P	pressure [MPa]
PCHE	printed circuit heat exchanger
PS	pump rotational speed [rpm]
sCO_2_	supercritical CO_2_
*T*	temperature [°C]
TG	turbogenerator
V	valve

## Declaration of competing interest

The authors declare that they have no conflicts of interest in this work.

## Appendix A On-road test data for the real-time vehicle engine

The input of the heat source should be determined by the real operation of the vehicle which is indispensable to representing the real on-road conditions of vehicles. In this study, the real-time on-road data obtained by vehicles from nine cities are used to select the boundary conditions of the experimental investigation.
